# Identifying definite patterns of unmet needs in patients with multiple sclerosis using unsupervised machine learning

**DOI:** 10.1007/s10072-024-07416-9

**Published:** 2024-02-23

**Authors:** Elisabetta Maida, Gianmarco Abbadessa, Eleonora Cocco, Paola Valentino, Annalaura Lerede, Jessica Frau, Giuseppina Miele, Floriana Bile, Marco Vercellino, Francesco Patti, Giovanna Borriello, Paola Cavalla, Maddalena Sparaco, Luigi Lavorgna, Simona Bonavita

**Affiliations:** 1 Department of Advanced Medical and Surgical Sciences, University of Campania “Luigi Vanvitelli”, Via Pansini 5, 80131 Naples, Italy; 2 Department of Brain Sciences, Imperial College London, London, W120BZ UK; 3 Department of Medical Science and Public Health, Centro Sclerosi Multipla, University of Cagliari, Cagliari, Italy; 4 Institute of Neurology, University Magna Graecia, Catanzaro, Viale Europa, Catanzaro, Italy; 5 MS Center, Department of Neuroscience, City of Health and Science University Hospital of Turin, Turin, Italy; 6 Department “GF Ingrassia”, Section of Neurosciences, University of Catania, Catania,, Italy; 7 MS Center, Hospital San Pietro Fatebenefratelli, Rome, Italy; 8 AOU Luigi Vanvitelli, Naples, Italy

**Keywords:** Unmet needs, Multiple sclerosis, Quality of life, Cluster

## Abstract

**Introduction:**

People with multiple sclerosis (PwMS) exhibit a spectrum of needs that extend beyond solely disease-related determinants. Investigating unmet needs from the patient perspective may address daily difficulties and optimize care. Our aim was to identify patterns of unmet needs among PwMS and their determinants.

**Methods:**

We conducted a cross-sectional multicentre study. Data were collected through an anonymous, self-administered online form. To cluster PwMS according to their main unmet needs, we performed agglomerative hierarchical clustering algorithm. Principal component analysis (PCA) was applied to visualize cluster distribution. Pairwise comparisons were used to evaluate demographics and clinical distribution among clusters.

**Results:**

Out of 1764 mailed questionnaires, we received 690 responses. Access to primary care was the main contributor to the overall unmet need burden. Four patterns were identified: cluster C1, ‘information-seekers with few unmet needs’; cluster C2, ‘high unmet needs’; cluster C3, ‘socially and assistance-dependent’; cluster C4, ‘self-sufficient with few unmet needs’. PCA identified two main components in determining the patterns: the ‘public sphere’ (access to information and care) and the ‘private sphere’ (need for assistance and social life). Older age, lower education, longer disease duration and higher disability characterized clusters with more unmet needs in the private sphere. However, demographic and clinical factors failed in explaining the four identified patterns.

**Conclusion:**

Our study identified four unmet need patterns among PwMS, emphasizing the importance of personalized care. While clinical and demographic factors provide some insight, additional variables warrant further investigation to fully understand unmet needs in PwMS.

**Supplementary Information:**

The online version contains supplementary material available at 10.1007/s10072-024-07416-9.

## Introduction

Multiple sclerosis (MS) is one of the most common neurological diseases impacting the central nervous system. Nowadays, MS is a major cause of permanent disability, contributing in 2016 to 0.04% of global disability-adjusted life-years (DALYs), with estimated 2.2 million cases worldwide [1]. As a chronic disease, MS requires a multifaceted interdisciplinary management, involving several clinical and administrative figures working together to guarantee the most appropriate path of care.

The term ‘unmet needs’ is used to describe ‘a situation in which individuals or groups fail to obtain benefits for various reasons, although they may do so from interventions or health service delivery’ [2]. The underlying nature of unmet needs is far from static, as it may undergo considerable changes according to the healthcare system and support services available in each country. The prevalence and perception of unmet needs among individuals with chronic diseases, such as MS, is likely to be significantly influenced by differences in access to services, healthcare policies and resource allocation across different countries.

People with MS (PwMS) might have various needs based on their disability, unique life experiences, individual traits and disease severity. Whenever these needs remain unaddressed, patients are left alone to struggle with the difficulties of their illness. Confronting with the unmet needs of PwMS should prompt to improve understanding and awareness about the patients’ perspective. Indeed, the investigation of unmet needs from the patients’ perspective can be useful to address daily difficulties and guiding the optimization of PwMS care. The final goal should be the implementation of an integrated, person-centred path of care [3].

To gain a more comprehensive understanding of unmet needs among PwMS, our objective is (i) to delineate the core patterns characterizing these needs. To achieve this, we employed a machine learning clustering approach, facilitating the categorization of individuals based on their reported unmet needs; (ii) to investigate whether these patterns are associated to specific sociodemographic and clinical features.

## Methods

### Study design

We conducted a cross-sectional multicentre study involving six specialized MS centres equally distributed from North to South on the Italian peninsula (Hospital San Pietro Fatebenefratelli, Rome; University ‘Magna Graecia’, Catanzaro; University of Catania, Catania; University of Cagliari, Cagliari; City of Health and Science University Hospital of Turin, Turin; University of Campania ‘Luigi Vanvitelli’, Naples). PwMS were invited to participate in the study by the Chief of each MS centre. An e-mail was sent to all PwMS within each MS centre, followed by a subsequent reminder midway through the study period. Data were collected from December 2022 to May 2023 through an anonymous online self-administered questionnaire, presented as a Google Form, and subsequent data were extrapolated for analysis. The study was conducted in accordance with the guidelines of the Declaration of Helsinki for human subjects’ research, and, at the beginning of the survey, the patient’s informed consent was obtained. The study was approved by the Ethical Committee of the University of Campania Luigi Vanvitelli (protocol number 0014460/i).

### Questionnaire

The full Italian (and the English translated) version of the questionnaire is reported in Supplementary.

Information about demographic status (gender, age, area of residence, education, living status and employment) and MS clinical features (disease duration; clinical phenotype—relapsing-remitting multiple sclerosis (RRMS), secondary-progressive multiple sclerosis (SPMS), primary-progressive multiple sclerosis (PPMS) and ‘I don’t know’—current and past disease-modifying therapy (DMT) and global disability) were collected. To assess PwMS’ disability, the Italian online version of the Patient Determined Disease Steps (PDDS) was employed [4], as it is a very intuitive self-administered disability scale highly correlated to the Expanded Disability Status Scale (EDSS).

The EQ-5D-5L (EuroQol-5 Dimension-5 Levels) questionnaire [5, 6] was used to explore perceived quality of life (QoL), as it measures health-related QoL (HRQoL). The test consists of a descriptive system and a visual analogue scale (EQ-5D VAS) which are administered together. The descriptive system includes five domains of everyday life (mobility, self-care, usual activities, pain/discomfort and anxiety/depression), each measured by five levels (1—no problem; 2—mild problems; 3—moderate problems; 4—severe problems; 5—extreme problems). The combined 5-value number represents the patient’s health status and is further converted into a utility index (with the maximum value being 1000, representing the best possible health) that is calculated from a population preference–based value set [7].

Finally, unmet needs were assessed with a 23-item questionnaire developed by the cooperation of a team of expert MS neurologists (S.B., E.M. and L.L.). The questionnaire was designed to examine different aspects of perceived comprehensive care and covered five domains such as ‘access to information’ (about MS features, therapeutic opportunities and the role of digital technologies) and ‘access to primary care’ (access to recommended therapies; to visits with specialists other than neurologists or to laboratory and imaging tests; to prescribed medical devices, physiotherapy and psychological support and to guaranteed government aid), ‘social life’ (with focus on social activities and participation in patient associations, satisfaction in physical well-being, or any discrimination experienced in everyday life), ‘need for assistance’ (in transportation, in daily activities, at work, and in their own homes) and finally ‘doctor-patient relationship’ (satisfaction in the relationship with the neurologist and any occasions when there were difficulties in communication or other issues). Every question within each domain allowed respondents to indicate whether the needs were satisfied (score of 0) or unmet (score of 1). Subsequently, we combined the scores from all questions, obtaining an overall score for each category (scores ranged between 0 and 3 for ‘access to information’, 0 and 7 for ‘access to primary care’, 0 and 4 for ‘social life’, 0 and 5 for the need for assistance and 0 and 4 for the doctor-patient relationship) and a total score (ranging between 0 and 23). An additional question investigated PwMS’ satisfaction with their treatment and focused on possible reasons for dissatisfaction (mode or frequency of administration, side effects, clinical worsening).

### Cluster and statistical analysis

Continuous variables were presented as mean and standard deviation (SD), while categorical variables were presented as number and percentage.

To assess which class of unmet needs contributes more significantly to the total score (total number of unmet needs), Spearman’s correlation coefficient was calculated between each class and total number of unmet needs.

The clustering variables were the five domains previously mentioned: access to information, access to primary care, social life, need for assistance, doctor-patient relationship. Our principal clustering algorithm was agglomerative hierarchical clustering (AHC), complemented by subsequent analyses employing k-means clustering techniques. Considering the characteristics of the clustering variables, which consist of count variables with varying range spans, we have applied a normalization approach to scale them. Subsequently, we conducted AHC using Ward’s method (specifically, Ward2 algorithm), with the similarity measure being defined by the Euclidean distance.

**Fig. 1:**
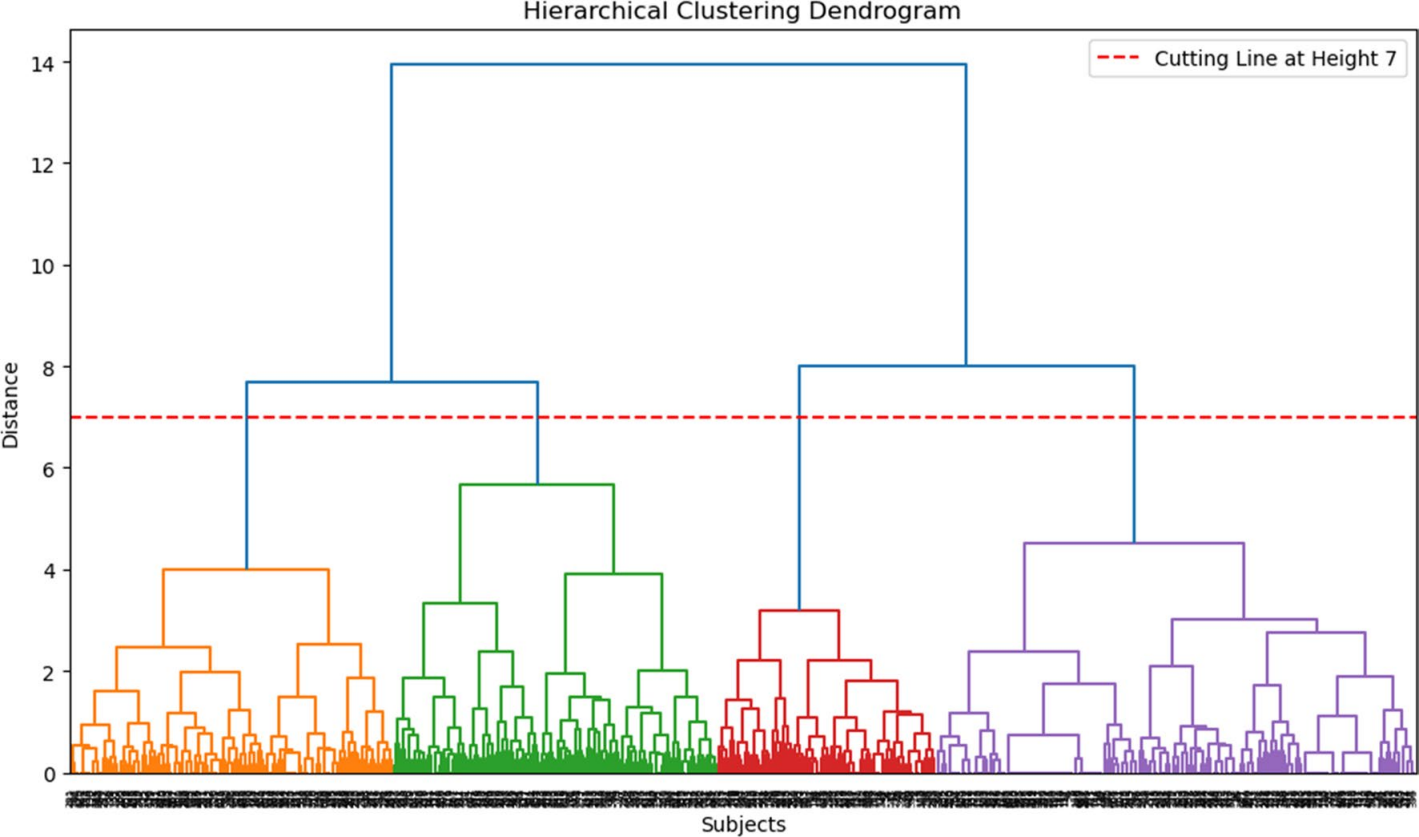
Hierarchical clustering dendrogram. The figure displays the hierarchical clustering dendrogram generated using the Ward linkage method. The dendrogram provides a visual representation of how the subjects in the dataset are grouped into clusters based on their similarities in the selected features. The *x*-axis represents the subjects. The *y*-axis represents the distance (dissimilarity) between clusters. The hierarchical structure of the dendrogram reveals the merging and splitting of clusters as the algorithm progresses. The vertical lines represent the fusion of clusters, with the height of each line indicating the dissimilarity at which the fusion occurred

To select the optimal number of clusters, we utilized two metrics: the silhouette score and the elbow method (Supplementary Figs. S1 and S2). Additionally, we examined the dendrogram structure (Fig. 1). Both metrics suggested that the ideal number of clusters was two. However, the silhouette score exhibited a range between 0.2 and 0.3 for cluster numbers ranging from two to five, suggesting a reasonable separation without clear dominance of any specific number of clusters. Based on the dendrogram structure and supported by the metrics, we decided to proceed with four clusters to explore the patterns of unmet needs within our population. Cluster labels were assigned by analysing the distributions of the cluster variables.

For dimensionality reduction, we utilized principal component analysis (PCA) with an initial component count set to five, equivalent to the number of patient experience variables. To determine the most representative number of components, we examined the eigenvalues on a scree plot and employed the ‘elbow’ method. As a result, two factors were determined to be optimal, a decision also substantiated by the Kaiser criterion (Supplementary Fig. S3). After identifying the optimal number of components, a PCA taking two components was performed, and a varimax rotation was applied to maximize the variance of the squared loadings. The loadings from this analysis depicted how the original variables contributed to the evaluated components. Lastly, a biplot was generated to visually represent the PCA results. This biplot displayed the scores of the observations on the principal components and the loadings of the variables.

To characterize the identified clusters, we assigned a label to each cluster and examined the differences in reported unmet needs among the clusters. Subsequently, we proceeded to describe and compare the demographic, social and clinical characteristics among the four clusters, to identify the factors characterizing the identified pattern. To compare categorical variables, we employed the chi-square test, while for continuous variables, we utilized the Kruskal-Wallis test. We conducted pairwise comparisons and adjusted the *p* values using the Bonferroni correction method.

## Results

### High prevalence of unmet needs among pwMS

A total of 1764 e-mails were sent from the six different centres located throughout the Italian peninsula. Six hundred ninety subjects answered the questionnaire in its entirety and were, therefore, included in the study, while an additional six individuals did not accept the privacy informed consent and were consequently excluded. The average response rate was 39.12%, in line with previous studies [8]. Mean age was 43.60 years (± 11.43 SD), and 70.14 % of subjects were female (*n* = 484). Demographic and disease features are reported in Table 1.

**Table 1:** Demographic and clinical characteristics (*N* = 690)

		Total (*n* = 690)
Age (y), mean (SD)		43.60 (11.43)
Female, *n* (%)		484 (70.14)
Education, *n* (%)	Primary School	102 (14.78)
	High School	345 (50.00)
	University degree	243 (35.22)
Geographical origin, *n* (%)	Northern Italy	139 (20.14)
	Centre Italy	112 (16.24)
	Southern Italy	225 (32.60)
	Island	214 (31.02)
Occupational status, *n* (%)	Currently employed	437 (63.33)
	Unemployed	253 (36.67)
Housing situation, *n* (%)	Living alone	86 (12.46)
	Living with someone	604 (87.54)
Duration of MS disease (y), mean (SD)		11.19 (8.89)
MS Form, *n* (%)	RR	500 (72.46)
	SP	63 (9.14)
	PP	29 (4.20)
	‘I don’t know’	98 (14.20)
Disability level, mean PDDS (SD)		2.04 (2.04)
DMTs modalities, *n* (%)	Oral	334 (48.40)
	Subcutaneous	106 (15.36)
	Intravenous	190 (27.54)
	Untreated	60 (8.70)
EQ-5D index utility scores, mean (SD)	*(Italian value set)*	0.722 (0.27)
EQ-5D VAS scores, mean (SD)		67.50 (20.74)

*y* years, *DMT* disease-modifying therapy, *MS* multiple sclerosis, *n* number, *PDDS* Patient-Determined Disease Steps Scale, *SD* standard deviation

Overall, 655 out of 690 PwMS (94.93 %) reported at least one need being unmet, with only 35 (5.07%) reporting no unmet needs; 400 out of 690 (58.08 %) reported having six or more unmet needs. Specifically, 65.80% and 69.28% showed one or more unmet need within the domain of access to information and access to primary care; 83.05% reported at least one unmet need in social life; 54.50% revealed at least one unmet need related to assistance; lastly, 37.40% reported one or more unmet need in doctor-patient relationship.

Among the five classes of unmet needs addressed by the questionnaire, difficulty in accessing primary care was found to be the major contributor to the total number of unmet needs (*R* = 0.744, *p* < 0.001), followed by social aspect (*R* = 0.718, *p* < 0.001) and the need for assistance and support (*R* = 0.663, *p* < 0.001). Correlation coefficients were interpreted as in Mukaka MM [9]. Spearman’s correlation results are reported in Table 2.

**Table 2:**
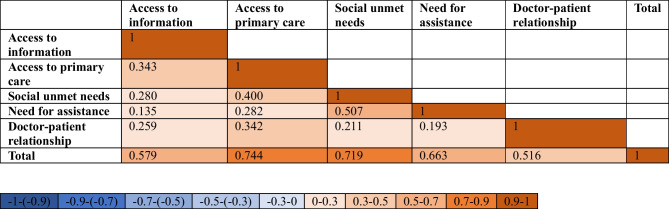
Spearman’s correlation. The heatmap depicts the direction of the correlation, with red tones trending towards a stronger association, negative or positive (± 1)

### Unmet needs in access to information, need for assistance and social life are key factors in defining cluster patterns

**Fig. 2:**
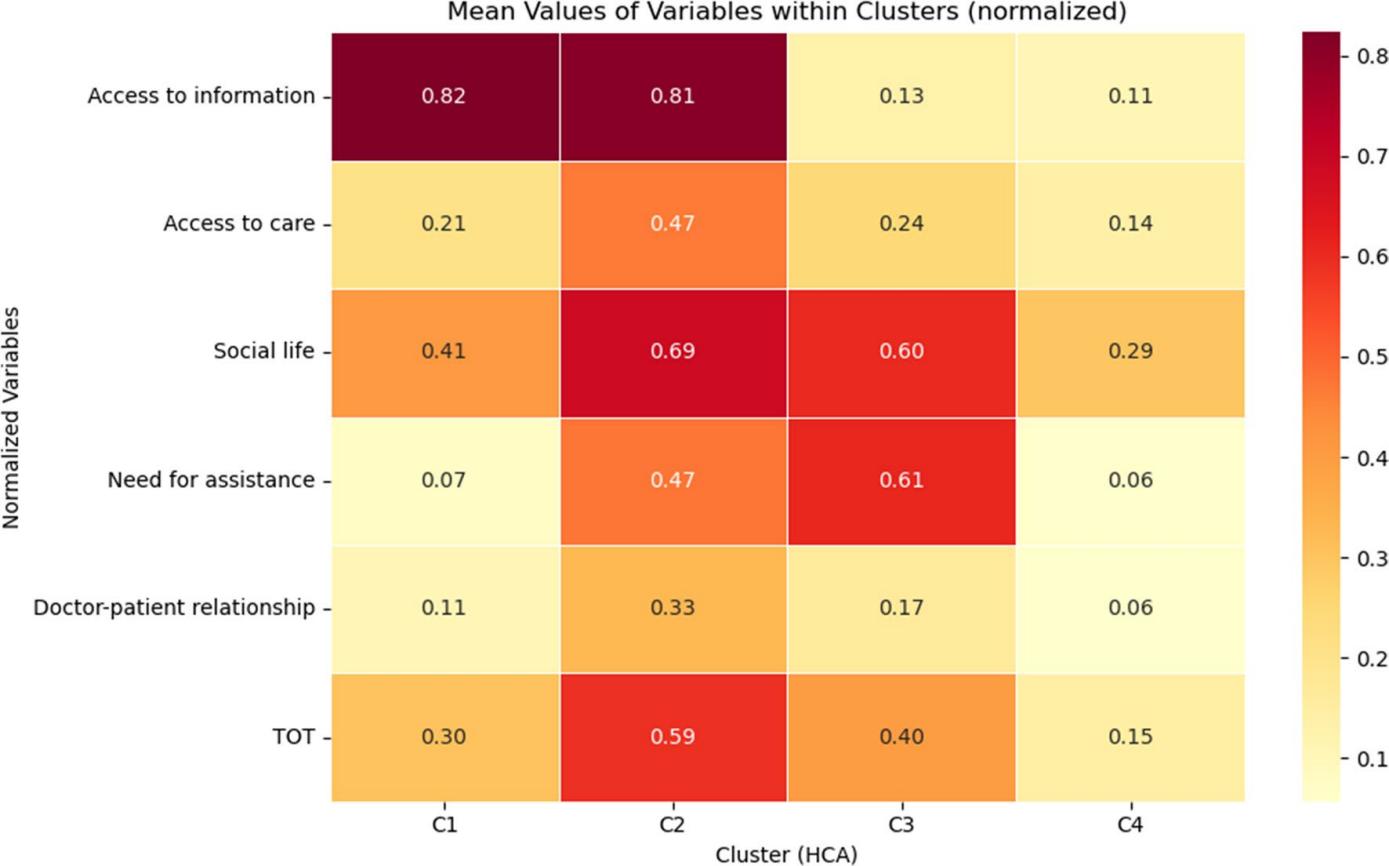
Mean values of normalized variables within clusters generated by hierarchical clustering algorithm (HCA). The heatmap presents the mean values of selected variables within distinct clusters, after the normalization process. The figure offers insights into how variables vary across different clusters. The *x*-axis represents the clusters: C1: cluster 1, C2: cluster 2, C3: cluster 3 and C4: cluster 4. The *y*-axis displays the variables used to cluster the subjects (‘access to information’, ‘access to care’, ‘social life’, ‘need for assistance’, ‘doctor-patient relationship’) and the total number of unmet needs (TOT). The colour intensity in each cell indicates the magnitude of the mean value, with a colour scale ranging from yellow (lower values) to red (higher values). Values are annotated within the cells for clarity. This visualization aids in identifying patterns, trends and differences among clusters based on the selected variables. It offers a comprehensive view of how these variables contribute to the characterization of each cluster, facilitating data-driven insights and decision-making

Four clusters were identified, labelled from C1 to C4 (Fig. 1). Cluster C1, referred to as ‘information-seekers with few unmet needs’, consisted of 166 patients (24.06%) and was characterized by a moderate to low prevalence of unmet needs in all domains, except that for a high number of unmet needs related to acquiring information concerning their condition. Cluster C2, named ‘high unmet needs’, was also composed of 166 patients (24.06%), and it was characterized by a high number of unmet needs in all the explored domains, with the highest score reached in access to information, access to care and social life. Cluster C3, ‘socially and assistance-dependent’ included 112 individuals (16.23 %) with significant unmet needs in self-care, autonomy and social life; conversely, subjects in this cluster reported only a moderate number of unmet needs in access to primary care and few unmet needs related to access to information. C4, self-sufficient with few unmet needs, consisted of 246 PwMS (35.65 %), which experienced no special struggles or unmet needs. Mean number of reported unmet needs for each domain within each cluster and pairwise comparisons between clusters with adjusted *p* values are reported in Supplementary Table S1. Overall, as also shown in the heatmap depicting the mean value of each domain within each cluster (Fig. 2), access to information, need for assistance and social life represent the prominent contributors to the cluster definition.

K-means clustering produced clusters that were overall similar to the hierarchical cluster analysis. The cluster sizes changed, with 206 individuals (29.86 %) in C1, 104 (15.07 %) in C2, 177 (25.65 %) in C3 and 203 (29.42 %) in C4. However, the mean value of the clustering variable within each cluster remained consistent, allowing the same labels (C1 to C4) to be applied as shown in Supplementary Fig. S4. Indeed, C1 encompassed subjects reporting exclusively a high number of unmet needs in access to information, while C2 consisted of subjects with the highest number of unmet needs in all the explored domains. In contrast, C3 reported difficulties in self-care, autonomy and social life. The only difference observed with this approach compared to AHC was that C3 reported a lower number of unmet needs for assistance compared to C2 (Supplementary Fig. S4).

**Fig. 3:**
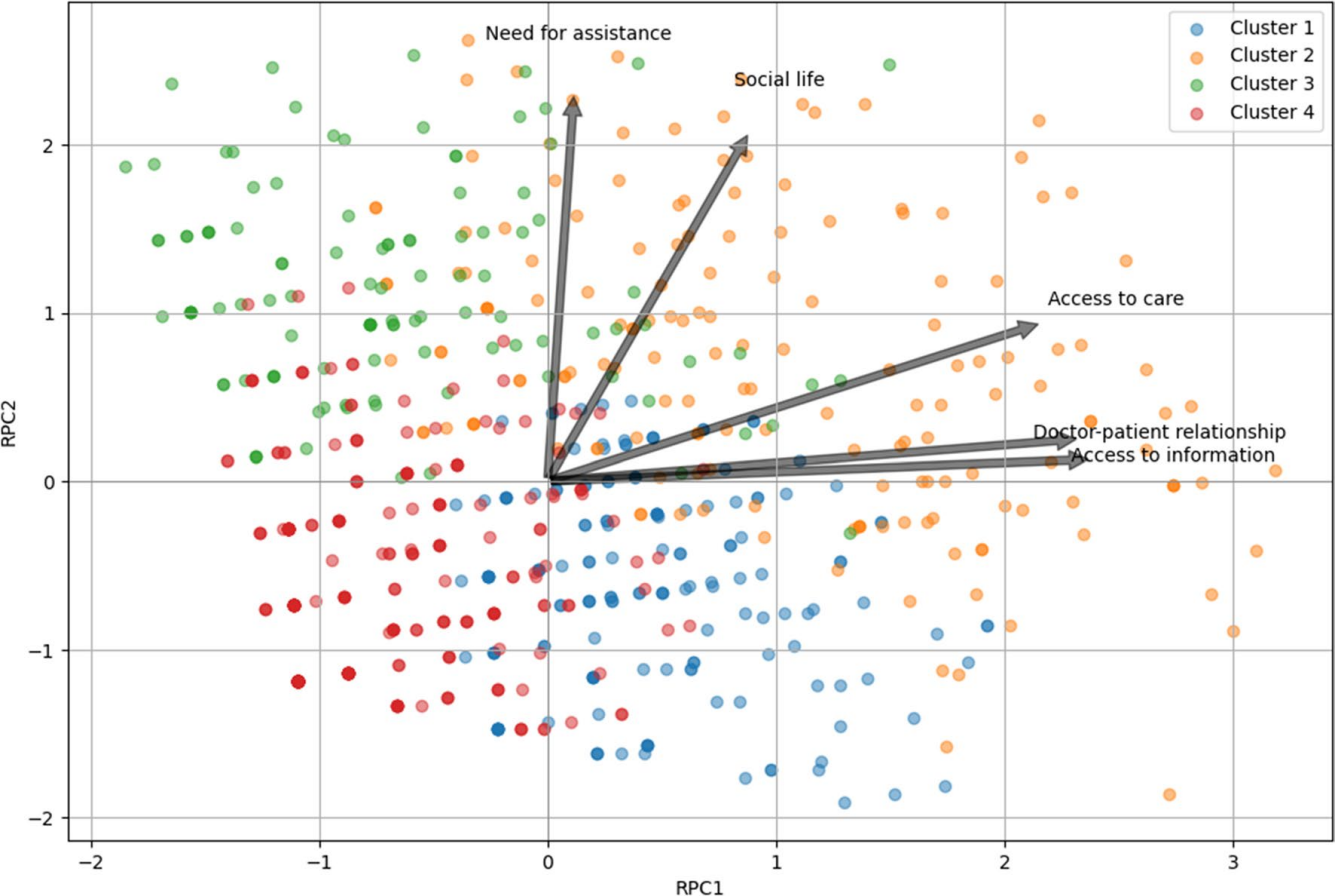
Biplots of principal component analysis (PCA) scores and component loadings with cluster assignments. A biplot showing both the PCA scores for individual data points and the PCA loadings for the selected variables. Data points are coloured based on their cluster assignments, with four distinct clusters being represented. The arrows represent the loadings of each selected variable on the two principal components, with the direction and length of each arrow indicating the direction and magnitude of each variable’s association with the components. The *x*-axis and *y*-axis represent the first and second rotated principal components (RPC1 and RPC2), respectively. The biplot aids in visualizing how clusters are separated in the PCA space and how each variable contributes to this separation

Finally, we conducted standard PCA to visualize the clusters (Fig. 3). Eigenvalue analysis indicated that two principal components explained 63.48% of the variance in the data and were optimal in representing the data. Analysis of the loadings, post varimax rotation, revealed the significance of each patient experience metric on the two principal components. The heatmap of factor loadings after varimax rotation (Supplementary Fig. S5) revealed that component 1 (PC1) predominantly relates to access to information, access to care, and the doctor-patient relationship, which might collectively be termed the public sphere. This component may reflect needs concerning support from external sources. Component 2 (PC2) focuses on the need for assistance (autonomy) and social life, aligning more with the private sphere. Looking at the biplot displaying the clusters’ distribution in a two-dimensional space (Fig. 3), we observed that C1 and C2 differ from other clusters mainly in PC1 (public sphere), while PC2 (private sphere) contributes more in differentiating C2 and C3 from C1 and C4, with individuals in the latter clusters having less needs related to the private sphere.

### Demographics and clinicals partially explain the identified patterns.

C2 and C3 showed a significantly higher mean age than C1 and C4. Individuals in C1 had a higher level of education than those in C2 and C3. The unemployment rate was relatively low for C1 and C4, while it was quite high in clusters 2 and 3, with nearly half of the population without a stable job.

C2 and C3 had longer disease duration and a higher degree of disability, measured by PDDS, than C1 and C4 (C1, 1.05 ± 1.20 SD; C2, 3.27 ± 2.07 SD; C3, 3.99 ± 1.95 SD; C4, 0.97 ± 1.22 SD). C2 and C3 had a significant higher prevalence of persons with a progressive disease (SPMS and PPMS) than C1 and C4.

In C1 and C4, more than half of the individuals were on oral disease-modifying therapy (DMTs). In contrast, the proportion of individuals receiving intravenous medication significantly rose in C2 and C3, along with the number of individuals not following any treatment. The majority of individuals in C1 and C4 were satisfied with their DMTs. In contrast, an opposite trend was observed in C2 and C3, with C2 resulting in the least satisfied group.

Lastly, QoL, as measured by both EQ-5D utility index and EQ-VAS, was significantly lower in C2 and C3, compared with C1 and C4. Demographic and disease characteristics per cluster and all pair-wise comparisons and adjusted *p* value are reported in Tables 3 and 4.

**Table 3:** Pairwise comparisons between each class of unmet need among each cluster

	Cluster 1 (C1) (*n* = 166)	Cluster 2 (C2) (*n* = 166)	Cluster 3 (C3) (*n* = 112)	Cluster 4 (C4) (*n* = 246)	*p* value (Bonferroni adjusted)
Access to information, mean (SD)	2.47 (0.55)	2.43 (0.69)	0.39 (0.49)	0.32 (0.46)	C4 vs C3 = 1.000000e + 00 **C4 vs C2 = 3.880957e − 66** **C4 vs C1 = 3.267655e − 70** **C3 vs C2 = 8.028977e − 43** **C3 vs C1 = 3.403062e − 46** C2 vs C1 = 1.000000e + 00
Access to primary care, mean (SD)	1.46 (1.38)	3.28 (1.89)	1.71 (1.59)	0.98 (1.233)	**C4 vs C3 = 4.312091e − 04** **C4 vs C2 = 6.861902e − 31** **C4 vs C1 = 3.736631e − 03** **C3 vs C2 = 6.679774e − 10** C3 vs C1 = 1.000000e + 00 **C2 vs C1 = 1.800593e − 16**
Social unmet needs, mean (SD)	1.63 (1.03)	2.75 (0.82)	2.41 (0.70)	1.18 (1.09)	**C4 vs C3 = 1.854751e − 20** **C4 vs C2 = 1.138622e − 34** **C4 vs C1 = 7.138794e − 04** **C3 vs C2 = 6.291820e − 03** **C3 vs C1 = 8.646531e − 09** **C2 vs C1 = 1.428214e − 19**
Need for assistance, mean (SD)	0.36 (0.51)	2.37 (1.64)	3.05 (1.17)	0.31 (0.54)	**C4 vs C3 = 1.474591e − 54** **C4 vs C2 = 1.399054e − 39** C4 vs C1 = 1.000000e + 00 **C3 vs C2 = 1.659834e − 02** **C3 vs C1 = 6.551035e − 45** **C2 vs C1 = 4.749897e − 30**
Doctor-patient relationship, mean (SD)	0.43 (0.72)	1.31 (1.15)	0.67 (0.90)	0.23 (0.51)	**C4 vs C3 = 3.414306e − 06** **C4 vs C2 = 2.248805e − 25** C4 vs C1 = 1.005513e − 01 **C3 vs C2 = 1.242660e − 04** C3 vs C1 = 6.304882e − 01 **C2 vs C1 = 3.160770e − 12**
Total number of unmet needs, mean (SD)	6.40 (2.34)	12.41 (3.37)	8.39 (2.96)	3.06 (2.28)	**C4 vs C3 = 1.161012e − 36** **C4 vs C2 = 1.052941e − 63** **C4 vs C1 = 2.948156e − 30** **C3 vs C2 = 2.858429e − 17** **C3 vs C1 = 2.820493e − 06** **C2 vs C1 = 2.672719e − 41**

*SD* standard deviation; the use of bold formatting within the table was employed to highlight significant values

**Table 4:** Pairwise comparisons between each variable among each cluster

		Cluster 1 (C1) (*n* = 166)	Cluster 2 (C2) (*n* = 166)	Cluster 3 (C3) (*n* = 112)	Cluster 4 (C4) (*n* = 246)	*p* value (Bonferroni adjusted)
Age (y), mean (SD)		42.28 (11.85)	46.29 (11.63)	46.39 (11.18)	42.45 (10.23)	C4 vs C3 = 5.767611e − 02 **C4 vs C2 = 2.779210e − 02** C4 vs C1 = 1.000000e + 00 C3 vs C2 = 1.000000e + 00 **C3 vs C1 = 4.069891e − 02** **C2 vs C1 = 2.992550e − 02**
Female, *n* (%)		128 (77.11)	114 (68.67)	80 (71.23)	162 (65.85)	C4 vs C3 = 2.135336 C4 vs C2 = 3.742896 C4 vs C1 = 0.114378 C3 vs C2 = 4.325223 C3 vs C1 = 2.116455 C2 vs C1 = 0.650922
Education, *n* (%)	Primary school	13 (7.83)	38 (22.89)	19 (16.96)	32 (13.01)	C4 vs C3 = 0.119220 C4 vs C2 = 0.057165 C4 vs C1 = 1.442909 C3 vs C2 = 1.507007 **C3 vs C1 = 0.033768** **C2 vs C1 = 0.002164**
	High school	85 (51.21)	80 (48.19)	65 (58.04)	115 (46.75)	
	University degree	68 (40.96)	48 (28.92)	28 (25.00)	99 (40.24)	
Geographical origin, *n* (%)	Northern Italy	38 (22.89)	29 (17.47)	24 (21.43)	48 (19.51)	C4 vs C3 = 0.230125 C4 vs C2 = 0.850711 C4 vs C1 = 3.233937 C3 vs C2 = 0.092149 **C3 vs C1 = 0.023420** C2 vs C1 = 0.333317
	Centre Italy	20 (12.05)	22 (13.25)	30 (26.79)	40 (16.26)	
	Southern Italy	47 (28.31)	69 (41.57)	34 (30.36)	75 (30.49)	
	Island	61 (36.75)	46 (27.71)	24 (21.43)	83 (33.74)	
Occupational status, *n* (%)	Currently employed	115 (69.28)	85 (51.20)	51 (45.54)	186 (75.61)	**C4 vs C3 = 2.904593e − 07** **C4 vs C2 = 3.179775e − 06** C4 vs C1 = 1.145537e + 00 C3 vs C2 = 2.524463e + 00 **C3 vs C1 = 7.569922e − 04** **C2 vs C1 = 6.873126e − 03**
	Unemployed	51 (30.72)	81 (48.80)	61 (54.46)	60 (24.39)	
Housing situation, *n* (%)	Living alone	22 (13.25)	23 (13.86)	10 (8.93)	31 (12.60)	C4 vs C3 = 2.429436 C4 vs C2 = 4.950275 C4 vs C1 = 5.790904 C3 vs C2 = 1.743967 C3 vs C1 = 2.156389 C2 vs C1 = 6.000000
	Living with someone	144 (86.75)	143 (86.14)	102 (91.07)	215 (87.40)	
MS disease duration (y), mean (SD)		9.38 (7.88)	12.54 (9.50)	14.68 (9.25)	10.65 (7.68)	**C4 vs C3 = 3.074736e − 03** C4 vs C2 = 1.000000e + 00 C4 vs C1 = 1.000000e + 00 C3 vs C2 = 8.082730e − 01 **C3 vs C1 = 2.001316e − 05** C2 vs C1 = 5.450631e − 02
MS phenotype, *n* (%)	RR	141 (84.94)	94 (56.63)	72 (64.29)	193 (78.46)	**C4 vs C3 = 7.762844e − 07** **C4 vs C2 = 1.154353e − 08** C4 vs C1 = 1.074776e + 00 C3 vs C2 = 2.870040e + 00 **C3 vs C1 = 2.289805e − 04** **C2 vs C1 = 4.917344e − 07**
	SP	6 (3.61)	31 (18.67)	20 (17.86)	6 (2.44)	
	PP	3 (1.81)	13 (7.83)	8 (7.14)	5 (2.03)	
	I don’t know	16 (9.64)	28 (16.87)	12 (10.71)	42 (17.07)	
Disability level, mean PDDS (SD)		1.05 (1.20)	3.28 (2.08)	3.99 (1.96)	0.96 (1.23)	C4 vs C3 = 7-908418e − 02 **C4 vs C2 = 3.175820e − 29** C4 vs C1 = 1.000000e + 00 C3 vs C2 = 1.061767e − 01 **C3 vs C1 = 5.725158e − 29** **C2 vs C1 = 1.225799e − 22**
DMTs administration modalities, *n* (%)	Oral	83 (50)	76 (45.78)	44 (39.29)	131 (53.25)	**C4 vs C3 = 0.003041** C4 vs C2 = 0.143119 C4 vs C1 = 4.614526 C3 vs C2 = 3.147529 **C3 vs C1 = 0.003078** C2 vs C1 = 0.064737
	Subcutaneous	35 (21.08)	18 (10.84)	11 (9.82)	42 (17.07)	
	Intravenous	39 (23.50)	54 (32.53)	39 (34.82)	58 (23.58)	
	Not in therapy	9 (5.42)	18 (10.85)	18 (16.07)	15 (6.10)	
DMTs satisfaction, *n* (%)	Satisfied	105 (63.25)	60 (36.14)	54 (48.65)	186 (75.61)	**C4 vs C3 = 5.702000e − 06** **C4 vs C2 = 1.567292e − 14** C4 vs C1 = 5.745257e − 02 C3 vs C2 = 3.086593e − 01 C3 vs C1 = 1.339430e − 01 **C2 vs C1 = 8.203713e − 06**
	Not satisfied	61 (36.75)	106 (63.86)	57 (51.35)	60 (24.39)	
EQ-5D index utility scores, mean (SD)	*(Italian value set)*	0.850 (0.13)	0.521 (0.33)	0.520 (0.29)	0.865 (0.13)	**C4 vs C3 = 3.629279e − 34** **C4 vs C2 = 1.240738e − 34** C4 vs C1 = 1.000000e + 00 C3 vs C2 = 1.000000e + 00 **C3 vs C1 = 1.242354e − 28** **C2 vs C1 = 1.753966e − 26**
EQ-5D VAS scores, mean (SD)		74.70 (15.23)	55.25 (20.85)	52.30 (19.56)	77.85 (15.44)	**C4 vs C3 = 1.455650e − 25** **C4 vs C2 = 1.973582e − 26** C4 vs C1 = 8.190953e − 01 C3 vs C2 = 1.000000e + 00 **C3 vs C1 = 2.820558e − 18** **C2 vs C1 = 4.495280e − 17**

*y* years, *DMT* disease-modifying therapy, *MS* multiple sclerosis, *PDDS* Patient-Determined Disease Steps Scale, *SD* standard deviation; the use of bold formatting within the table was employed to highlight significant values

## Discussion

Providing proper and constant care for PwMS presents several challenges due to the variability of clinical features at disease onset and along disease evolution, which often leads to several needs being unmet. In our study, we comprehensively assessed demographic and clinical characteristics of PwMS and their unmet needs in Italy to (i) provide a snapshot of PwMS unmet needs in Italy; (ii) identify distinct clusters of subjects according to the unmet needs and their main determinants and (iii) evaluate how demographics and clinical factors were distributed among these clusters.

A significant finding in our study was that nearly all the patients reported unmet needs, highlighting potential deficiencies in the healthcare system’s ability to manage a chronic disease such as MS. The most pronounced category of unmet needs was related to difficulties in accessing care, closely followed by social aspects and the need for assistance in daily life. This limited accessibility to healthcare services poses significant challenges in obtaining entitled care, often necessitating out-of-pocket expenses for patients. Additionally, our research suggests a pervasive sense of social inadequacy among PwMS. Therefore, neurologists should encourage PwMS to maintain their routines and introduce them to tailored exercise programs to boost their self-confidence in their physical well-being [10]. In summary, we observed a high prevalence of unmet needs among PwMS, with the primary care domain being the primary contributor to the overall burden. This may underscore the shortcomings in the Italian healthcare system in managing chronic diseases.

The unsupervised clustering approach identified four main patterns of unmet needs among PwMS. These differed from each other in the total number and typology of reported unmet needs. C1 and C4 emerged as the clusters with the fewest unmet needs. Specifically, PwMS belonging to C4 had not reported significant unmet needs in any of the five categories. In C1, PwMS referred exclusively a high number of unmet needs in the domain of access to information. C2 appeared to be the cluster with the highest cumulative number of unmet needs, especially in the domain of access to information and social life. Finally, C3 included individuals reporting an overall moderate number of unmet needs with the highest score in need for assistance and social life. The domains that more contributed to cluster definition were access to information, need for assistance and social life. This finding should prompt MS neurologists to carefully investigate these domains to provide a more personalized approach in MS management.

Moreover, PCA enabled us to condense our variables into two primary components, indicating that they can be categorized into two spheres: the public sphere (pertaining the access to information and access to primary care) and the private sphere (related to disability and social life). Among the demographic and clinical variables examined, there was a marked distinction between clusters C2 and C3, which were characterized by a higher prevalence of unmet needs (especially in the private sphere) and a lower QoL, compared to clusters C1 and C4, which reported fewer unmet needs. The populations of C2 and C3 predominantly consisted of older individuals, who typically had lower education levels and faced higher unemployment rates. In terms of disease characteristics, the individuals in C2 and C3 exhibited longer disease duration, greater disability and higher frequency of subjects reporting a progressive phenotype, compared to those in C1 and C4. This observation is in line with existing research [11], highlighting that older age, lower educational attainment, extended disease duration and higher disability levels are critical determinants of reduced QoL and more frequent unmet needs. As it is plausible that a better clinical condition may alleviate some needs, it is essential to address the potential disparities in care for patients with more severe conditions. Doctors should play a key role in bridging this gap by implementing personalized treatment strategies and providing comprehensive support services.

However, demographic and clinical factors alone do not fully elucidate the four distinct patterns identified. Indeed, no significant demographic, social or clinical variable differences were found when comparing C1 with C4 or C2 with C3. In summary, factors such as older age, lower education level, longer disease duration and higher disability predominantly characterize clusters with a moderate to high range of unmet needs, especially in domains like assistance requirement and social life (private sphere). However, these factors fail to fully explain the varying patterns observed, as they do not account for differences in unmet needs pertaining to the public sphere.

This observation warrants particular emphasis, as it demonstrates that this approach has provided a level of granularity that allows for the identification of unmet need patterns not accounted for by conventional clinical, demographic and disease-related variables. Indeed, our findings have revealed that regardless of motor disability and other collected information, there are patients with specific unresolved needs. The rationale for the similarity between these two pairs of clusters (C1–C4 and C2–C3) may be attributed to the presence of additional variables that were not investigated in the present study. These could include cognitive status, depressive or anxious symptoms, possible comorbidities and lifestyle factors related to the environment and economic status. Consequently, it is imperative for future research to further explore the underlying factors contributing to the differences among the clusters, thus allowing for a more accurate definition. This update will enable highly customized unmet needs resolution strategies based on individual characteristics.

Another interpretation suggests that the two clusters with a high degree of unmet needs may represent a temporal progression from one of the clusters with a low degree of unmet needs. For example, C2 may arise from C1 and C3 may arise from C4. Although this hypothesis cannot be confirmed, the particular distribution of unmet needs categories suggests its plausibility. Future longitudinal studies may be able to provide further details on this connection and potential predictive factors related to the transition between clusters. For example, a recurring theme for clusters C1 and C2 is the high number of unmet needs in the category of access to information. This finding should prompt neurologists to increase the engagement of their patients by (1) improving doctor-patient communication to enable PwMS to be fully informed and active in healthcare decisions; (2) increasing awareness of symptoms, especially the ‘hidden‘ ones and (3) providing greater emphasis on patient-reported outcomes (PROs) to establish a patient-centred approach [12, 13]. These results can be achieved by devoting more effort to informing patients, organizing in-person or telematic meetings and instructing PwMS how to use properly the resources available online.

This study provided an updated overview of the unmet needs of Italian PwMS by considering a broad and widely heterogeneous sample. Our findings showed that demographic and clinical variables only partially explain the observed patterns. Indeed, despite sociodemographics and clinicals differ between patients with a higher number of unmet needs in the private sphere (autonomy and social life), they do not allow to discriminate the observed heterogenous patterns. Therefore, the neurologist should pay special attention to the presence of specific unmet needs in all PwMS regardless of the presence or not of unfavourable sociodemographic and clinical features. This approach will provide a deep understanding of each individual’s specific needs, facilitating an effective and personalized approach to address the needs and improve the overall QoL of PwMS.

Limitations include not having incorporated the rural-urban area differentiation or socioeconomic status and not having assessed the cognitive status and depressive or anxiety symptoms that might have influenced the results. Indeed, more studies are needed to investigate additional variables that were not investigated by the present research. Furthermore, Google Form lacks a feature to detect duplicate entries without compromising respondent anonymity. Despite recognizing this constraint, we prioritized respondent anonymity over the possibility of excluding duplicates. However, since the length of a questionnaire can impact response rates, we believed that only a very small number of users would attempt to respond multiple times [14, 15]. Finally, having employed e-mail and digital media as a means of recruiting PwMS may have limited the study population, thus creating a selection bias. Therefore, as the mean response rate is 39.12%, it is possible that the study only partially reflects the entire population of PwMS.

## Conclusion

Our study highlights the significant prevalence of unmet needs among PwMS, pointing to potential shortcomings within the healthcare system in managing chronic diseases such as MS. We identified four distinct patterns of unmet needs, underscoring the necessity of tailoring care to each patient’s specific requirements. While clinical and demographic factors offer some insight, unexplored variables, including cognitive status and socioeconomic factors, could play a role in shaping unmet needs and should be further explored in future research. Addressing these unmet needs on an individual basis has the potential to significantly improve the overall quality of life of PwMS.

### Supplementary Information

Below is the link to the electronic supplementary material.

Supplementary file1 (DOCX 210 KB)

## Data Availability

The data that support the findings of this study are available from the corresponding author, SB, upon reasonable request.

## References

[1] GBD (2016). Multiple Sclerosis Collaborators (2019) Global, regional, and national burden of multiple sclerosis 1990–2016: a systematic analysis for the Global Burden of Disease Study 2016. Lancet Neurol.

[2] Harrison JD, Young JM, Butow PN, Solomon MJ (2013). Needs in health care: what beast is that?. Int J Health Serv.

[3] World Health Organization (2022) https://www.who.int/news/item/27-05-2022-seventy-fifth-world-health-assembly-daily-update27-may-2022. Accessed 15 Nov 2023

[4] Lavorgna L, Miele G, Petruzzo M, Lanzillo R, Bonavita S (2018). Online validation of the Italian version of the patient determined disease steps scale (PDDS) in people with multiple sclerosis. Mult Scler Relat Disord.

[5] EuroQol Group (1990). EuroQol-a new facility for the measurement of health-related quality of life. Health Policy.

[6] Herdman M, Gudex C, Lloyd A, Janssen M, Kind P, Parkin D, Bonsel G, Badia X (2011). Development and preliminary testing of the new five-level version of EQ-5D (EQ-5D-5L). Qual Life Res.

[7] Finch AP, Meregaglia M, Ciani O, Roudijk B, Jommi C (2022). An EQ-5D-5L value set for Italy using videoconferencing interviews and feasibility of a new mode of administration. Soc Sci Med.

[8] Meng-Jia W, Kelly Z, Francisca FA (2022) Response rates of online surveys in published research: a meta-analysis. Comput Hum Behav 100206 10.1016/j.chbr.2022.100206

[9] Mukaka MM (2012). Statistics corner: a guide to appropriate use of correlation coefficient in medical research. Malawi Med J.

[10] Kalb R, Brown TR, Coote S, Costello K, Dalgas U, Garmon E, Giesser B, Halper J, Karpatkin H, Keller J, Ng AV, Pilutti LA, Rohrig A, Van Asch P, Zackowski K, Motl RW (2020). Exercise and lifestyle physical activity recommendations for people with multiple sclerosis throughout the disease course. Mult Scler.

[11] Ponzio M, Tacchino A, Zaratin P, Vaccaro C, Battaglia MA (2015). Unmet care needs of people with a neurological chronic disease: a cross-sectional study in Italy on Multiple Sclerosis. Eur J Public Health.

[12] Rieckmann P, Boyko A, Centonze D, Elovaara I, Giovannoni G, Havrdová E, Hommes O, Kesselring J, Kobelt G, Langdon D, LeLorier J, Morrow SA, Oreja-Guevara C, Schippling S, Thalheim C, Thompson H, Vermersch P (2015). Achieving patient engagement in multiple sclerosis: a perspective from the multiple sclerosis in the 21st Century Steering Group. Mult Scler Relat Disord.

[13] Rieckmann P, Centonze D, Elovaara I, Giovannoni G, Havrdová E, Kesselring J, Kobelt G, Langdon D, Morrow SA, Oreja-Guevara C, Schippling S, Thalheim C, Thompson H, Vermersch P, Aston K, Bauer B, Demory C, Giambastiani MP, Hlavacova J, Nouvet-Gire J, Pepper G, Pontaga M, Rogan E, Rogalski C, van Galen P, Ben-Amor AF (2018). Unmet needs, burden of treatment, and patient engagement in multiple sclerosis: a combined perspective from the MS in the 21st Century Steering Group. Mult Scler Relat Disord.

[14] Gummer T, Roßmann J (2015). Explaining interview duration in web surveys: a multilevel approacH. Soc Sci Comput Rev.

[15] Fan W, Yan Z (2010). Factors affecting response rates of the web survey: a systematic review. Comput Hum Behav.

