# Supplementary material for: Identifying definite patterns of unmet needs in patients with multiple sclerosis using unsupervised machine learning
Source: Neurol Sci. 2024 Feb 23;45(7):3333–45. doi: 10.1007/s10072-024-07416-9 (PMC11176240; doi:10.1007/s10072-024-07416-9)
Supplement: Supplementary file 1 — Supplementary file1 (DOCX 210 KB) [file 10072_2024_7416_MOESM1_ESM.docx]

**Identifying definite patterns of unmet needs in patients with Multiple Sclerosis using unsupervised machine learning**

**Neurological Sciences**

Elisabetta Maida^1#^, Gianmarco Abbadessa^1,2#^, Eleonora Cocco^3^, Paola Valentino^4^, Annalaura Lerede^2^, Jessica Frau^3^, Giuseppina Miele^1^, Floriana Bile^1^, Marco Vercellino^5^, Francesco Patti^6^, Giovanna Borriello^7^, Paola Cavalla^5^, Maddalena Sparaco^8^, Luigi Lavorgna^8^, Simona Bonavita^1*^

*^1^Department of Advanced Medical and Surgical Sciences, University of Campania “Luigi Vanvitelli”, Naples, Italy*

*^2^Department of Brain Sciences, Imperial College London, London W120BZ, UK*

*^3^Department of Medical Science and Public health, Centro Sclerosi Multipla, University of Cagliari, Cagliari, Italy.*

*^4^Institute of Neurology, University Magna Graecia, Catanzaro, Viale Europa, Catanzaro, Italy*

*^5^MS Center, Department of Neuroscience, City of Health and Science University Hospital of Turin, Turin, Italy*

*^6^Department "GF Ingrassia", Section of Neurosciences, University of Catania, Catania, Italy*

*^7^MS Center, Hospital San Pietro Fatebenefratelli, Rome, Italy.*

*^8^AOU Luigi Vanvitelli, Naples, Italy*

^#^These authors contributed equally.

***Corresponding author:**

**Simona Bonavita**

Department of Advanced Medical and Surgical Sciences, University of Campania Luigi Vanvitelli, Via Pansini 5, 80131, Naples, Italy

Tel: +39 081 566 6742; e-mail: simona.bonavita@unicampania.it

**Supplementary Material**

**Full Questionnaire (original Italian version)**

Demografica

**Q1**. Data di nascita

GG/MM/AAAA

**Q2**. Sesso

- M
- F
- Preferisco non dire

**Q3**. In che regione vive?

- Abbruzzo
- Basilicata
- Calabria
- Campania
- Emilia-Romagna
- Friuli-Venezia Giulia
- Lazio
- Liguria
- Lombardia
- Marche
- Molise
- Piemonte
- Puglia
- Sardegna
- Sicilia
- Toscana
- Trentino-Alto Adige
- Umbria
- Valle d’Aosta
- Veneto

**Q4**. Che scuole ha completato?

- Elementari
- Medie
- Liceo o istituto professionale
- Laurea triennale
- Laurea magistrale
- Specializzazione/Master/Dottorato

**Q5**. Stato abitativo

*Selezionare anche più di una risposta*

- Convivo con il/la partner/coniuge
- Vivo con i miei genitori
- Vivo da solo/a
- Vivo con i miei figli

**Q6**. Lavoro

- Lavoro a tempo pieno
- Lavoro part-time
- La mia disabilità mi permette di lavorare solo part-time
- Disoccupato/a
- La mia disabilità non mi permette di lavorare
- Pensionato/a
- Casalingo/a
- Indichi la sua situazione:
- Dipendente
- Libero professionista
- Svolgo sia attività pubblica che privata

Informazioni di malattia

**Q1**. Anno della diagnosi

*In caso non ricordasse precisamente giorno e mese inserisca 15/06/aaaa*

GG/MM/AAAA

**Q2**. Forma di malattia come definita dal mio neurologo

- Recidivante-Remittente
- Secondariamente Progressiva
- Primariamente Progressiva
- Non so

**Q3**. Leggendo le seguenti descrizioni, scelga quella che descrive meglio la sua condizione. Con questa scala vogliamo capire la sua capacità di camminare. Potrebbe, quindi, non trovare una descrizione che rifletta esattamente la sua condizione; in questo caso scelga la categoria che più si avvicina alla sua situazione

- 0 - Nessuna disabilità. A volte mi capita di avere qualche sintomo legato alla Sclerosi Multipla, per lo più riguardante la sensibilità, ma questo non limita le mie normali attività. Se ho una ricaduta, tutto ritorna alla normalità quando il sintomo sparisce.
- 1 - Disabilità lieve: ho qualche sintomo evidente della Sclerosi Multipla che, però, interferisce poco con il mio stile di vita.
- 2 - Disabilità moderata: non ho nessuna limitazione nel camminare; tuttavia, ho dei problemi dipendenti dalla Sclerosi Multipla che limitano la mia vita quotidiana ma non sono legati alla deambulazione.
- 3 - Disabilità nel camminare: La Sclerosi Multipla limita la mia vita quotidiana, specialmente la mia capacità di camminare. Riesco a lavorare ma se devo impegnarmi in attività fisiche o sportive ho molte difficoltà rispetto ad una volta. Cammino senza appoggio ma durante le ricadute della malattia ho bisogno di assistenza.
- 4 - Singolo appoggio in fase iniziale: Uso un bastone o una sola stampella o altre forme di appoggio (ad esempio mi appoggio al muro o al braccio di qualcuno) per camminare tutto il tempo o la maggior parte del tempo, specialmente negli spazi esterni. Credo di riuscire a camminare per 7.5mt in 20 secondi senza il bastone o la stampella. Ho sempre bisogno di supporto (bastone o stampella) se voglio camminare per 3 isolati (circa 100 mt)
- 5 - Singolo appoggio in fase avanzata: per camminare 7.5 metri devo avere o un bastone, o una stampella o qualcuno a cui appoggiarmi. Posso girare per casa o in altri ambienti appoggiandomi ai mobili o alle pareti. Se voglio fare percorsi più lunghi utilizzo la sedia a rotelle o la sedia a rotelle motorizzata
- 6 - Doppio appoggio: per camminare 7.5 metri devo avere o due bastoni, o due stampelle o un deambulatore. Utilizzo la sedia a rotelle o la sedia a rotelle motorizzata per distanze più lunghe
- 7 - Sedia a rotelle/sedia a rotelle motorizzata: la mia principale modalità di spostamento è la sedia a rotelle. Sono capace di alzarmi e fare uno o due passi ma non posso camminare per 7.5 metri anche con le stampelle o il deambulatore
- 8 - Costretto a letto: incapace di stare sulla sedia a rotelle per più di un’ora.

**Q4**. Terapia attuale

- Aubagio
- Avonex
- Betaferon
- Copaxone
- Copemyl/Copemyltri
- Extavia
- Gylenia
- Kesimpta
- Lemtrada
- Mavenclad
- Mayzent
- Ocrevus
- Plegridy
- Ponvory
- Rebif
- Tecfidera
- Tysabri
- Vumerity
- Zeposia
- Altro
- Attualmente non sono in terapia

**Q5**. Quanto sei soddisfatto della tua terapia?

- Molto
- Poco
- Sono mediamente soddisfatto

🡪Indichi le ragioni per cui non si ritiene soddisfatto della sua attuale terapia:

*Selezionare anche più di una risposta*

- Non mi piace la modalità di somministrazione
- Non mi piace la frequenza di somministrazione
- Mi da troppi effetti collaterali
- Mi sento comunque peggiorare nella mia sintomatologia

**Q6**. Quali farmaci ha assunto dalla sua diagnosi?

*Selezionare anche più di una risposta*

- Aubagio
- Avonex
- Betaferon
- Copaxone
- Copemyl/Copemyltri
- Extavia
- Gylenia
- Kesimpta
- Lemtrada
- Mavenclad
- Mayzent
- Ocrevus
- Plegridy
- Ponvory
- Rebif
- Tecfidera
- Tysabri
- Vumerity
- Zeposia
- Non ricordo

La sua qualità di vita

**Q1**. Capacità di movimento

- Non ho difficoltà nel camminare
- Ho lievi difficoltà nel camminare
- Ho moderate difficoltà nel camminare
- Ho gravi difficoltà nel camminare
- Non sono in grado di camminare

**Q2**. Cura della persona

- Non ho difficoltà nel lavarmi o vestirmi
- Ho lievi difficoltà nel lavarmi o vestirmi
- Ho moderate difficoltà nel lavarmi o vestirmi
- Ho gravi difficoltà nel lavarmi o vestirmi
- Non sono in grado di lavarmi o vestirmi

**Q3**. Attività abituali (per es. lavoro, studio, lavori domestici, attività familiari o di svago)

- Non ho difficoltà nello svolgimento delle attività abituali
- Ho lievi difficoltà nello svolgimento delle attività abituali
- Ho moderate difficoltà nello svolgimento delle attività abituali
- Ho gravi difficoltà nello svolgimento delle attività abituali
- Non sono in grado di svolgere le mie attività abituali

**Q4**. Dolore o fastidio

- Non provo alcun dolore o fastidio
- Provo lieve dolore o fastidio
- Provo moderato dolore o fastidio
- Provo grave dolore o fastidio
- Provo estremo dolore o fastidio

**Q5**. Ansia o depressione

- Non sono ansioso/a o depresso/a
- Sono lievemente ansioso/a o depresso/a
- Sono moderatamente ansioso/a o depresso/a
- Sono gravemente ansioso/a o depresso/a
- Sono estremamente ansioso/a o depresso/a

**Q6**. Vorremmo sapere quanto è buona o cattiva la sua salute oggi.

*In una scala numerata che va da 0 a 100, dove 100 rappresenta la migliore salute che può immaginare, mentre 0 rappresenta la peggiore salute che può immaginare. Scriva nella casella qui sotto il numero che meglio rappresenta la sua salute oggi.*

Cosa le manca

**Q1**. Ho accesso a tutte le informazioni che vorrei riguardo la Sclerosi Multipla (tramite il neurologo, i social media, le campagne di sensibilizzazione, ecc…)

- Si
- No, vorrei essere informato/a di più
- Non mi interessa o non mi sono mai informato/a

**Q2**. Ho accesso a tutte le informazioni che vorrei riguardo le diverse possibilità terapeutiche per la Sclerosi Multipla (compreso i nuovi farmaci che saranno presto immessi in commercio)

- Si
- No, vorrei essere informato/a di più
- Non mi interessa o non mi sono mai informato/a

**Q3**. Sono a conoscenza del ruolo delle tecnologie digitali nella gestione della Sclerosi Multipla (Visite in remoto, App, Social Media, “Terapie Digitali”, ecc…)

- Si
- No, vorrei essere informato/a di più
- Non mi interessa o non mi sono mai informato/a

**Q4**. Ho avuto facile accesso alle terapie prescritte dal neurologo (farmaci specifici per la Sclerosi Multipla o farmaci per i sintomi)

- Si
- No

**Q5**. Ho avuto facile accesso a visite presso gli altri medici specialisti e personale sanitario che intervengono nella cura della Sclerosi Multipla (Urologo, Ginecologo, Psicologo, Fisiatra, Fisioterapista, Nutrizionista, ecc…)

- Si
- No
- Non mi interessa o non mi sono mai informato/a

**Q6**. Ho avuto facile accesso ai dispositivi medici prescritti dallo specialista (sedia a rotelle, stampelle, presidi assorbenti, tutori, ecc…)

- Si
- No
- Non mi interessa o non mi sono mai informato/a

**Q7**. Ho avuto facile accesso alla terapia riabilitativa (fisioterapia) in convenzione con il SSN

- Si
- No
- Non mi interessa o non mi sono mai informato/a

**Q8**. Sono riuscito/a ad effettuare facilmente esami (esami del sangue, risonanza) per il controllo della Sclerosi Multipla

- Si
- No

**Q9**. Ho avuto facile accesso ad un supporto psicologico professionale

- Si
- No
- Non mi interessa o non mi sono mai informato/a

**Q10**. Sono riuscito/a a richiedere ed ottenere facilmente aiuti statali per la disabilità (INPS)

- Si
- No
- Non mi interessa o non mi sono mai informato/a

**Q11**. Sono membro di associazioni di pazienti, gruppi di supporto, ecc…

- Si
- No, ma vorrei
- Non mi interessa o non mi sono mai informato/a
- Le trova utili?
- Si
- No

**Q12**. Sono soddisfatto/a della mia vita sociale (hobbies, uscite di piacere, relazioni sociali)

- Si, la Sclerosi Multipla non influenza in alcun modo la mia vita sociale
- Così così, la Sclerosi Multipla talora influenza la mia vita sociale
- No, la Sclerosi Multipla influenza completamente la mia vita sociale

**Q13**. Sono soddisfatto/a del mio livello di attività fisica e sport

- Si, la Sclerosi Multipla non influenza in alcun modo la mia attività fisica
- Così così, la Sclerosi Multipla talora influenza la mia attività fisica
- No, la Sclerosi Multipla influenza completamente la mia attività fisica

**Q14**. Riesco a svolgere tutte le mansioni richieste dal mio lavoro

- Si
- No, la Sclerosi Multipla mi limita in alcune cose
- No, non riesco a lavorare per la Sclerosi Multipla
- Al momento sono disoccupato

**Q15**. Ho necessità di aiuto negli spostamenti quotidiani (andare a lavoro, a fare la spesa, a fare fisioterapia o altri controlli medici)

- Si
- No

**Q16**. Ho necessità di aiuto nelle attività della vita quotidiana (lavarsi, vestirsi, mangiare, etc...)?

- Si
- No
- A volte
- La figura che l’aiuta
- È una figura professionale a mie spese
- Assistenza domiciliare integrata a carico del Servizio Sanitario Nazionale
- È un familiare/amico
- Alterno a seconda delle possibilità

**Q17**. Ho avuto difficoltà a causa delle barriere architettoniche

- Si
- No

**Q18**. Ho avuto necessità di adattare la mia abitazione per la Sclerosi Multipla

- Si
- No

**Q19**. Ho subito discriminazioni per via della malattia

- Si
- No
- In che ambiente

*Selezionare anche più di una risposta*

- Familiare
- Amicizie
- Lavorativo

**Q20**. Sono soddisfatto/a del rapporto con il mio neurologo

- Si
- No

🡪 Perché?

*Selezionare anche più di una risposta*

- È emotivamente distante
- Non è abbastanza disponibile o non è facilmente raggiungibile
- Non mi dedica il tempo che vorrei durante la visita
- Non mi informa adeguatamente rispetto alle scelte da intraprendere
- Fornisce spiegazioni in un linguaggio troppo tecnico da comprendere
- Non prende sul serio quello che gli riferisco
- Altro

**Q21**. Ho mai avuto difficoltà nel comunicare da remoto con il mio neurologo?

- Sì; non risponde quasi mai a mail, chiamate, messaggi, ecc…
- A volte; risponde tardi o in maniera poco esaustiva
- No; non ho mai avuto problemi

**Q22**. C’è stato qualche momento nella tua storia clinica con la sclerosi multipla in cui hai ritenuto il tuo neurologo inadeguato?

*Selezionare anche più di una risposta*

- Al momento della diagnosi
- Nel seguirmi con scrupolo nel corso della malattia
- Nel comunicarmi che la terapia stava andando male
- Non è capitato in alcuna occasione

**Q23**. Il mio orientamento sessuale è:

- Eterosessuale
- Omosessuale
- Altro
- Non voglio rispondere

**Q24**. Mi sono mai sentito/a discriminata dal mio neurologo per il mio orientamento sessuale o non mi sono sentito/a libero/a di manifestare il mio orientamento sessuale

- Si
- No
- Qualche volta

**Full Questionnaire (translated English version)**

Demographic

**Q1.** Date of birth

DD/MM/YYYY

**Q2.** Gender

- Male
- Female
- Prefer not to say

**Q3**. What region do you live in?

- Abruzzo
- Basilicata
- Calabria
- Campania
- Emilia-Romagna
- Friuli-Venezia Giulia
- Lazio
- Liguria
- Lombardia
- Marche
- Molise
- Piemonte
- Puglia
- Sardegna
- Sicilia
- Toscana
- Trentino-Alto Adige
- Umbria
- Valle d’Aosta
- Veneto

**Q4**. What level of education have you completed?

- Elementary school
- Middle school
- High school or vocational school
- Bachelor's degree
- Master's degree
- Specialization/Master's/Ph.D.

**Q5**. Living situation

*Select all that apply*

- Living with my partner/spouse
- Living with my parents
- Living alone
- Living with my children

**Q6.** Employment

- Full-time employment
- Part-time employment
- My disability allows me to work only part-time
- Unemployed
- My disability prevents me from working
- Retired
- Homemaker

🡪 Please indicate your employment status:

- Employee
- Self-employed
- Engage in both public and private sector work

Health Information

**Q1.** Year of diagnosis

*If you don't remember the exact day and month, please use 15/06/YYYY.*

DD/MM/YYYY

**Q2.** Disease type as defined by my neurologist

- Relapsing-Remitting
- Secondary Progressive
- Primary Progressive
- I don't know

**Q3.** Please read the choices listed below and choose the one that best describes your own situation.This scale focuses mainly on how well you walk. You might not find a description that reflects your condition exactly, but please mark the one category that describes your situation the closest.

- 0 - normal: I may have some mild symptoms, mostly sensory due to MS but they do not limit my activity. If I do have an attack, I return to normal when the attack has passed.
- 1 - Mild disability: I have some noticeable symptoms from my MS but they are minor and have only a small effect on my lifestyle.
- 2 - Moderate disability: I don't have any limitations in my walking ability. However, I do have significant problems due to MS that limit daily activities in other ways.
- 3 - Gait disability: MS does interfere with my activities, especially my walking. I can work a full day, but athletic or physically demanding activities are more difficult than they used to be. I usually don't need a cane or other assistance to walk, but I might need some assistance during an attack.
- 4 - Early cane: I use a cane or a single crutch or some other form of support (such as touching a wall or leaning on someone's arm) for walking all the time or part of the time, especially when walking outside. I think I can walk 25 feet in 20 seconds without a cane or crutch. I always need some assistance (cane or crutch) if I want to walk as far as 3 blocks.
- 5 - Late cane: To be able to walk 25 feet, I have to have a cane, crutch or someone to hold onto. I can get around the house or other buildings by holding onto furniture or touching the walls for support. I may use a scooter or wheelchair if I want to go greater distances.
- 6 - Bilateral support: To be able to walk as far as 25 feet I must have 2 canes or crutches or a walker. I may use a scooter or wheelchair for longer distances.
- 7 - Wheelchair/scooter: My main form of mobility is a wheelchair. I may be able to stand and/or take one or two steps, but I can't walk 25 feet, even with crutches or a walker.
- 8 - Bedridden: Unable to sit in a wheelchair for more than one hour.

**Q4.** Current therapy

- Aubagio
- Avonex
- Betaferon
- Copaxone
- Copemyl/Copemyltri
- Extavia
- Gylenia
- Kesimpta
- Lemtrada
- Mavenclad
- Mayzent
- Ocrevus
- Plegridy
- Ponvory
- Rebif
- Tecfidera
- Tysabri
- Vumerity
- Zeposia
- Other
- Currently not on therapy

**Q5.** How satisfied are you with your current therapy?

- Very satisfied
- Not very satisfied
- Moderately satisfied

🡪 Please indicate the reasons for your dissatisfaction with your current therapy:

*Select all that apply*

- I don't like the mode of administration
- I don't like the frequency of administration
- It gives me too many side effects
- I still feel my symptoms are worsening

**Q6.** Which medications have you taken since your diagnosis?

*Select all that apply*

- Aubagio
- Avonex
- Betaferon
- Copaxone
- Copemyl/Copemyltri
- Extavia
- Gylenia
- Kesimpta
- Lemtrada
- Mavenclad
- Mayzent
- Ocrevus
- Plegridy
- Ponvory
- Rebif
- Tecfidera
- Tysabri
- Vumerity
- Zeposia
- I don't remember

Your Quality of Life

**Q1.** Mobility

- I have no problems in walking about
- I have slight problems in walking about
- I have moderate problems in walking about
- I have severe problems in walking about
- I am unable to walk about

**Q2.** Self-care

- I have no problems washing or dressing myself
- I have slight problems washing or dressing myself
- I have moderate problems washing or dressing myself
- I have severe problems washing or dressing myself
- I am unable to wash or dress myself

**Q3.** Usual activities (e.g., work, study, housework, family or leisure activities)

- I have no problems doing my usual activities
- I have slight problems doing my usual activities
- I have moderate problems doing my usual activities
- I have severe problems doing my usual activities
- I am unable to do my usual activities

**Q4.** Pain/discomfort

- I have no pain or discomfort
- I have slight pain or discomfort
- I have moderate pain or discomfort
- I have severe pain or discomfort
- I have extreme pain or discomfort

**Q5.** Anxiety/depression

- I am not anxious or depressed
- I am slightly anxious or depressed
- I am moderately anxious or depressed
- I am severely anxious or depressed
- I am extremely anxious or depressed

**Q6.** We would like to know how good or bad your health is today.

*On a scale from 0 to 100, where 100 means the best health you can imagine, and 0 means the worst health you can imagine, please write the number that best represents your health today in the box below.*

Your unmet needs

**Q1.** I have access to all the information I want regarding Multiple Sclerosis (through my neurologist, social media, awareness campaigns, etc.).

- Yes

- No, I would like to be more informed

- I'm not interested or have never sought information

**Q2.** I have access to all the information I want regarding the various MS treatment options (including new drugs soon to be on the market).

- Yes

- No, I would like to be more informed

- I'm not interested or have never sought information

**Q3.** I am aware of the role of digital technologies in MS management (remote consultations, apps, social media, "Digital Therapies," etc.).

- Yes

- No, I would like to be more informed

- I'm not interested or have never sought information

**Q4.** I have had easy access to the treatments prescribed by the neurologist (specific MS drugs or symptom-relief medications).

- Yes

- No

**Q5.** I have had easy access to appointments with other specialist doctors and healthcare professionals involved in MS care (urologist, gynecologist, psychologist, physiatrist, physiotherapist, nutritionist, etc.).

- Yes

- No

- I'm not interested or have never sought information

**Q6.** I have had easy access to medical devices prescribed by the specialist (wheelchair, crutches, absorbent aids, braces, etc.).

- Yes

- No

- I'm not interested or have never sought information

**Q7.** I have had easy access to rehabilitative therapy (physical therapy) covered by the National Health Service (NHS).

- Yes

- No

- I'm not interested or have never sought information

**Q8.** I have been able to easily undergo tests (blood tests, MRI) for MS monitoring.

- Yes

- No

**Q9.** I have had easy access to professional psychological support.

- Yes

- No

- I'm not interested or have never sought information

**Q10.** I have been able to easily request and receive state assistance for disability (INPS).

- Yes

- No

- I'm not interested or have never sought information

**Q11.** I am a member of patient associations, support groups, etc.

- Yes

- No, but I would like to be

- I'm not interested or have't explored this option

🡪 Do you find them helpful?

- Yes

- No

**Q12.** I am satisfied with my social life (hobbies, leisure outings, social relationships).

- Yes, MS does not influence my social life in any way

- So-so, MS sometimes affects my social life

- No, MS completely affects my social life

**Q13.** I am satisfied with my level of physical activity and sports.

- Yes, MS does not affect my physical activity in any way

- So-so, MS sometimes affects my physical activity

- No, MS completely affects my physical activity

**Q14.** I can perform all the tasks required by my job.

- Yes

- No, MS limits me in some things

- No, I cannot work due to MS

- I am currently unemployed

**Q15.** I need assistance in daily transportation (going to work, shopping, attending physiotherapy or other medical appointments).

- Yes

- No

**Q16.** I need assistance in daily life activities (bathing, dressing, eating, etc.).

- Yes

- No

- Sometimes

🡪 The person who assists me:

- A professional caregiver at my expense

- Integrated home care covered by the National Health Service

- A family member/friend

- Alternates depending on availability

**Q17.** I have faced difficulties due to architectural barriers.

- Yes

- No

**Q18.** I have needed to adapt my home for MS.

- Yes

- No

**Q19.** I have experienced discrimination because of my disease.

- Yes

- No

🡪 In what environment

*Select all that apply*

- Family

- Friendships

- Workplace

**Q20.** I am satisfied with my relationship with my neurologist.

- Yes

- No

🡪 Why?

*Select all that apply*

- They are emotionally distant

- They are not sufficiently available or easily reachable

- They do not dedicate the time I desire during appointments

- They do not provide adequate information regarding treatment choices

- They use overly technical language that is difficult to understand

- They do not take what I report seriously

- Other

**Q21.** Have you ever had difficulty communicating remotely with your neurologist?

- Yes; they rarely respond to emails, calls, messages, etc.

- Sometimes; they respond late or with insufficient information

- No; I have never had any issues

**Q22.** Have you ever found your neurologist inadequate in your MS history?

*Select all that apply*

- At the time of diagnosis

- In closely monitoring me during the course of the disease

- In communicating that the treatment was not going well

- It has not occurred on any occasion

**Q23.** My sexual orientation is:

- Heterosexual

- Homosexual

- Other

- I prefer not to answer

**Q24.** Have I ever felt discriminated against by my neurologist because of my sexual orientation or felt unable to express my sexual orientation freely?

- Yes

- No

- Sometimes

Domains of unmet needs (question within each domain)

Access to information: Q1-Q3

Access to primary care: Q4-Q10

Social life: Q11-Q13, Q19

Need for assistance: Q14-Q18

Doctor-patient relationship: Q20-Q22; Q24

**Table S1.** Pairwise Comparisons of Clustering Variables Between Four Clusters of pwMS.

|  | **Cluster 1 (C1)**  **(n=166)** | **Cluster 2 (C2)**  **(n=166)** | **Cluster 3 (C3)**  **(n=112)** | **Cluster 4 (C4)**  **(n=246)** | **p-value (Bonferroni adjusted)** |
| --- | --- | --- | --- | --- | --- |
| **Access to information, mean (SD)** | 2.47 (0.55) | 2.43 (0.69) | 0.39 (0.49) | 0.32 (0.46) | C4 vs C3=1.000000e+00  **C4 vs C2=3.880957e-66**  **C4 vs C1=3.267655e-70**  **C3 vs C2=8.028977e-43**  **C3 vs C1=3.403062e-46**  C2 vs C1=1.000000e+00 |
| **Access to primary care, mean (SD)** | 1.46 (1.38) | 3.28 (1.89) | 1.71 (1.59) | 0.98 (1.233) | **C4 vs C3=4.312091e-04**  **C4 vs C2=6.861902e-31**  **C4 vs C1=3.736631e-03**  **C3 vs C2=6.679774e-10**  C3 vs C1=1.000000e+00  **C2 vs C1=1.800593e-16** |
| **Social life, mean (SD)** | 1.63 (1.03) | 2.75 (0.82) | 2.41 (0.70) | 1.18 (1.09) | **C4 vs C3=1.854751e-20**  **C4 vs C2=1.138622e-34**  **C4 vs C1=7.138794e-04**  **C3 vs C2=6.291820e-03**  **C3 vs C1=8.646531e-09**  **C2 vs C1=1.428214e-19** |
| **Need for assistance, mean (SD)** | 0.36 (0.51) | 2.37 (1.64) | 3.05 (1.17) | 0.31 (0.54) | **C4 vs C3=1.474591e-54**  **C4 vs C2=1.399054e-39**  C4 vs C1=1.000000e+00  **C3 vs C2=1.659834e-02**  **C3 vs C1=6.551035e-45**  **C2 vs C1=4.749897e-30** |
| **Doctor-patient relationship, mean (SD)** | 0.43 (0.72) | 1.31 (1.15) | 0.67 (0.90) | 0.23 (0.51) | **C4 vs C3=3.414306e-06**  **C4 vs C2=2.248805e-25**  C4 vs C1=1.005513e-01  **C3 vs C2=1.242660e-04**  C3 vs C1=6.304882e-01  **C2 vs C1=3.160770e-12** |
| **Total number of unmet needs, mean (SD)** | 6.40 (2.34) | 12.41 (3.37) | 8.39 (2.96) | 3.06 (2.28) | **C4 vs C3=1.161012e-36**  **C4 vs C2=1.052941e-63**  **C4 vs C1=2.948156e-30**  **C3 vs C2=2.858429e-17**  **C3 vs C1=2.820493e-06**  **C2 vs C1=2.672719e-41** |

SD: standard deviation.


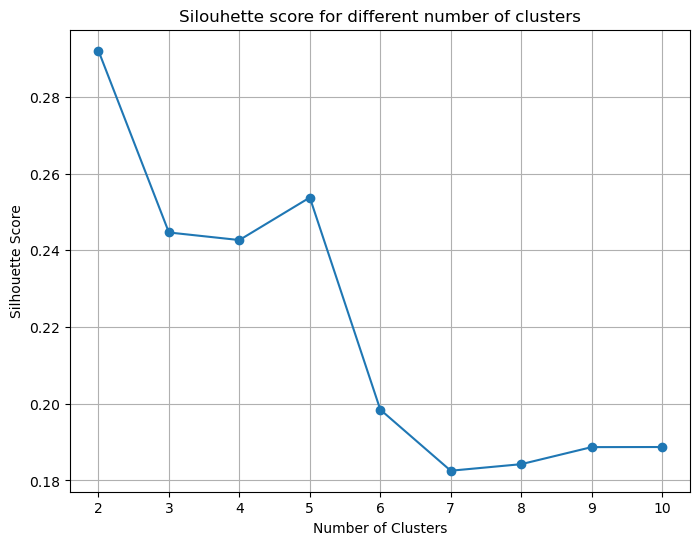


**Figure S1** Silhouette score for different number of clusters. The figure illustrates the relationship between the number of clusters and the corresponding silhouette scores in the hierarchical clustering analysis of the dataset. The silhouette score measures the quality of clustering, with higher scores indicating better separation between clusters. By providing a measure of how close each point in one cluster is to points in the neighboring clusters, this score ranges from -1 to 1. A higher silhouette score indicates better-defined clusters.


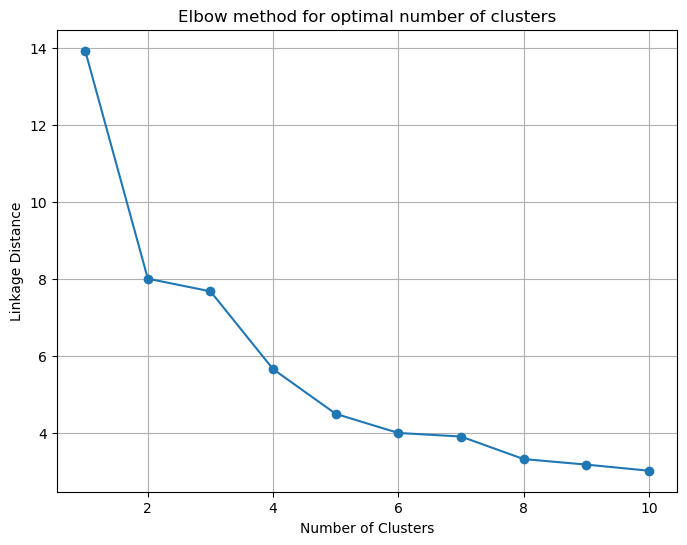


**Figure S2** Elbow method for optimal number of clusters (Ward Linkage). The figure illustrates the results of the Elbow Method analysis to determine the optimal number of clusters in the hierarchical clustering of the dataset. The analysis is performed using Ward linkage, a method for hierarchical clustering. The x-axis represents different numbers of clusters (ranging from 1 to 10), while the y-axis shows the linkage distances associated with each number of clusters. Linkage distances represent the dissimilarity between clusters, and they are calculated based on the Ward linkage method. The Elbow method plots the total within-cluster sum of squares against the number of clusters. The aim is to find the point where adding another cluster does not give much better modelling of the data – this point is known as the 'elbow'.

**
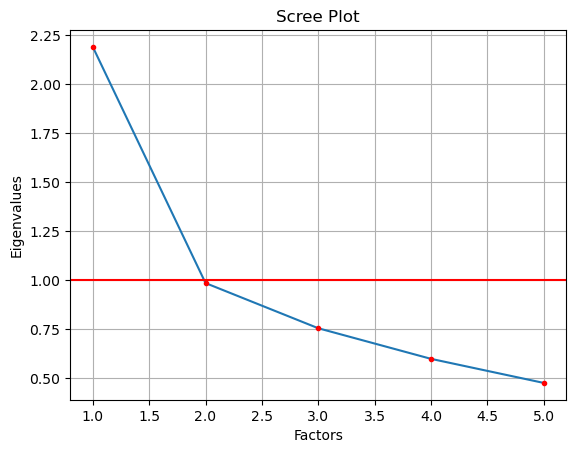
**

**Figure S3** Scree plot for optimal number of components. Scree plot illustrating eigenvalues against the number of factors. The plot suggests the inclusion of two factors, as indicated by the eigenvalues greater than or close to the red horizontal line (cutoff at eigenvalue = 1).


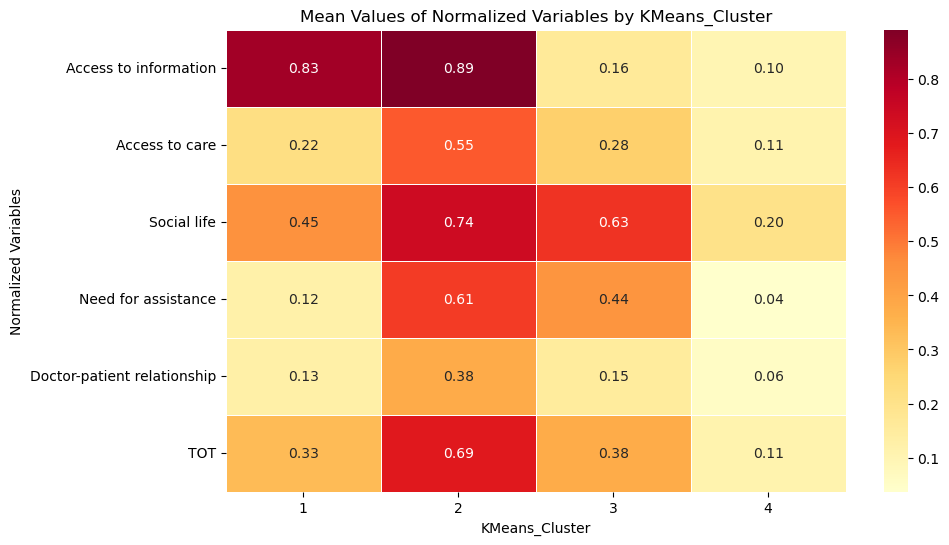


**Figure S4** Mean values of normalized variables within clusters generated by k-means. The heatmap presents the mean values of selected variables within distinct clusters, after the normalization process. The figure offers insights into how variables vary across different clusters. The x-axis represents the clusters: C1: Cluster 1, C2: Cluster 2, C3: Cluster 3 and C4: Cluster4. The y-axis displays the variables used to cluster the subjects (“Access to information”, “Access to care”, “Social life”, “Need for assistance”, “Doctor-patient relationship”) and the total number of unmet needs (TOT). The colour intensity in each cell indicates the magnitude of the mean value, with a colour scale ranging from yellow (lower values) to red (higher values). Values are annotated within the cells for clarity. This visualization aids in identifying patterns, trends, and differences among clusters based on the selected variables. It offers a comprehensive view of how these variables contribute to the characterization of each cluster, facilitating data-driven insights and decision-making.


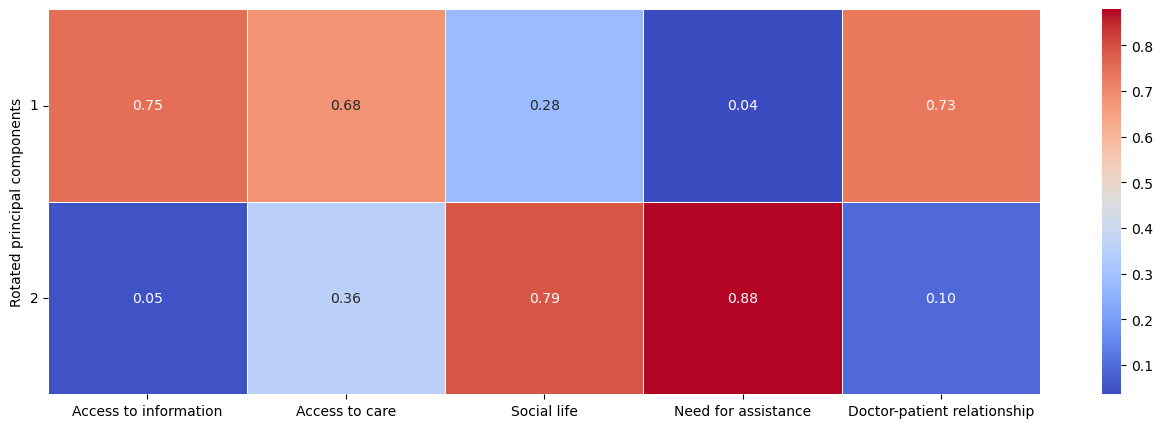


**Figure S5.** Factor loadings of rotated principal components (RPC) on selected variables. Heatmap representation of the factor loadings derived from the principal component analysis with varimax rotation. The two rotated principal components (RPC1 and RPC2) are displayed on the y-axis, while the selected variables, including access to information, access to care, social life, need for assistance, and doctor-patient relationship, are shown on the x-axis. The color gradient, ranging from blue to red, represents the strength of the loading, with values closer to 1 or -1 indicating stronger associations. Numeric values within the cells provide the exact loading of each variable on the respective component.
